# Geographic variation in the PRNP gene and its promoter, and their relationship to chronic wasting disease in North American deer

**DOI:** 10.1080/19336896.2020.1796250

**Published:** 2020-07-26

**Authors:** Robert M. Zink, Nadje Najar, Hernán Vázquez-Miranda, Brittaney L. Buchanan, Duan Loy, Bruce W. Brodersen

**Affiliations:** a School of Natural Resources, University of Nebraska-Lincoln, Lincoln, NE, USA; b School of Biological Sciences, University of Nebraska-Lincoln, Lincoln, NE, USA; c Nebraska State Museum, University of Nebraska-Lincoln, Lincoln, NE, USA; d Departamento de Zoología, Instituto de Biología, Universidad Nacional Autónoma de México, Ciudad de México, CP, Mexico; e Veterinary Diagnostic Center, School of Veterinary Medicine and Biomedical Sciences, University of Nebraska-Lincoln, Lincoln, NE, USA

**Keywords:** Coues deer, genotype, Key deer, mule deer, octarepeats, prion, promoter indels, white-tailed deer

## Abstract

PRNP genotypes, number of octarepeats (PHGGGWGQ) and indels in the PRNP promoter can influence the progression of prion disease in mammals. We found no relationship between presence of promoter indels in white-tailed deer and mule deer from Nebraska and CWD presence. White-tailed deer with the 95 H allele and G20D mule deer were more likely to be CWD-free, but unlike other studies white-tailed deer with the 96S allele(s) were equally likely to be CWD-free. We provide the first information on PRNP genotypes and indels in the promoter for Key deer (all homozygous 96SS) and Coues deer (lacked 95 H and 96S alleles, but possessed a uniquely high frequency of 103 T). All deer surveyed were homozygous for three tandem octarepeats.

## Introduction

Chronic Wasting Disease (CWD), a transmissible spongiform encephalopathy (TSE) of cervids, has led to declines of mule deer (*Odocoileus hemionus*) and white-tailed deer (*O. virginianus*) over the last two decades [1,2]. An incomplete understanding of the disease and its genetic control has in part hampered attempts to manage outbreaks. The relationship between different genotypes at the PRNP gene and the expression of CWD in cervids was explored in several studies [3–10]. For example, Johnson et al. [8] and Race et al. [10] provided experimental evidence that white-tailed deer orally inoculated with prions from CWD-positive deer expressing 95 H surviving the longest post-inoculation. Mule deer with at least one F at position 225 lived longer than wild type deer [11]. Thus, there is evidence that an individual’s PRNP genotype can influence the rate at which an infected deer succumbs to CWD. Unlike the situation with scrapie in sheep [12], there does not appear to be a genotype that provides nearly complete resistance to CWD in deer, although Haley et al. [13] suggest there might be. Vázquez-Miranda and Zink [14] pointed out that a single mutation in the PRNP gene in white-tailed deer could yield a genotype similar to the one in sheep that provides resistance to scrapie.

Variation in the coding region of PRNP is not the only genetic factor that can influence the expression of TSEs. In humans, variation in the number of octarepeat units (PHGGGWGQ) in the PRNP gene affects the frequency of prion disease [15], and polymorphisms in this repeat also affect progression of scrapie in sheep and goats [16]. In contrast, point mutations in PRNP are not correlated to reduced TSE susceptibility in bovids, whereas a 23-bp deletion in the PRNP promoter region on at least one allele is associated with increased susceptibility to bovine spongiform encephalopathy in some cattle breeds and the gayal (*Bos frontalis*) [17–22] Prion gene polymorphisms that affect BSE differ between cattle and buffalo [23]. Comparatively little is known about the association between polymorphisms in the PRNP promoter and the PrP protein, deletions in the promoter, frequency of octarepeats, and the expression of CWD in cervids. Heaton et al. [24] compared variation in the PRNP gene and the PRNP third exon that includes susceptibility codons and its likely promoter region [25] across sheep, beef cattle and deer, and found 21 polymorphic sites in 50 white-tailed deer and 43 mule deer from Wyoming sampled from 1996 to 1998. The 21 sites found in the deer PRNP promoter region included an eight-base indel. Heaton et al. [24] pointed out that these polymorphisms present an opportunity to test for association with CWD susceptibility. The PRNP promoter region has been characterized in elk (*Cervus elaphus*), a species known to have differentially susceptible PRNP genotypes [26] without also testing for CWD prevalence in relation to genotype [27]. To date, we are unaware of studies that compared PRNP genotypes and deletions in the promoter to deer of known CWD status.

The University of Nebraska-Lincoln Veterinary Diagnostic Center tested lymph nodes of 1807 white-tailed deer and mule deer harvested by hunters in 2017 and found that 203 (11.2%) were positive for CWD [28]. Individuals were scored as CWD+ based on their ELISA OD values. We sequenced the PRNP gene and the same promoter region as sampled by Heaton et al. [24] for most of the 203 infected deer and an approximately equal number of randomly chosen non-infected individuals from each species to explore the association between PRNP genotypes, presence of deletions in the promoter and the presence of CWD. Because no information on the incidence of indels in the promoter exists for other deer, we sequenced these two genes for small samples of white-tailed deer from Minnesota and New York, Florida’s endangered Key deer (*O. v. clavium*), Coues deer from Arizona (*O. v. couesi*), and black-tailed deer from California (*O. h. columbianus*) and Alaska (*O. h. sitkensis*). However, we lacked information on the presence of CWD in these deer, although CWD is currently absent from Key deer and Coues deer. Lastly, we comment on the existence and polymorphisms of octarepeats in deer PRNP because they have been implicated in prion disease in reindeer (*Rangifer tarandus*) [29] and other species [15,16].

## Results

We identified 11 PRNP genotypes for white-tailed deer and mule deer from Nebraska (Table 1). The wild type (WT) genotype (DQGASQ/DQGASQ) occurred in the majority of individuals in both species (mule deer: 102, white-tailed deer: 122) and was split uniformly between those with and without CWD (Table 1). Several genotypes were too rare to calculate odds ratios on. In mule deer, the G20D polymorphism was significantly associated with reduced odds of being CWD positive, whereas codon F225S polymorphisms had no effect. Mule deer were homozygous at all other loci. In white-tailed deer, H95Q was found in only nine individuals but was nearly significantly associated with reduced odds of contracting CWD (odds ratio 0.13, P = 0.051). G116A increased the odds of contracting CWD, but not significantly so (odds ratio 1.85, P = 0.332); polymorphisms at 96 and 226 appeared to have no effect. Genotypic data for 229 white-tailed deer and 137 mule deer in Nebraska for which we lacked information on CWD status closely matched the results reported here [14]. We identified two mule deer with an apparently novel allele for the species (K226Q); both were heterozygous (226Q/226 K). This allele has previously been reported in white-tailed deer at low frequencies [24]. Based on genotypes, we identified three putative F1 hybrids between mule deer and white-tailed deer.

**Table 1.** PRNP amino acid genotypes and presence of CWD in white-tailed deer and mule deer from Nebraska. Wt = wild type. Amino acid positions: 20, 95, 96, 116, 225 and 226.

	# CWD positive		# CWD negative		Totals	
Genotype	O.v.	O. h.	O. v.	O. h.	O. v.	O. h.
DQGASQ/DQGASQ (wt)	59	53	61	47	120	100
DQSASQ/DQGASQ	25	0	24	0	49	0
GQGASQ/DQGASQ	0	6	0	11	0	17
DQGGSQ/DQGASQ	6	0	3	0	9	0
DQGAFQ/DQGASQ	0	6	0	6	0	12
GQGASQ/GQGASQ	0	1	0	3	0	4
DQSASQ/DQSASQ	5	0	5	0	10	0
GQGASQ/DGGASQ	0	0	0	2	0	2
DHGASQ/DQGASQ	1	0	8	0	9	0
DQGASK/DQGASQ	1	0	1	0	2	0
DQSGSQ/DQGASQ	1	0	1	0	2	0
GQGACQ/DQGASQ	0	0	0	1	0	1

A total of 20 white-tailed deer possessed one of two deletions in the promoter region, including seven individuals that were heterozygous and one that was homozygous for the 8-base pair deletion found by Heaton et al. [24], and 11 individuals that were heterozygous and two that were homozygous for a 4-base deletion that has not to our knowledge been reported (Table 2). One white-tailed deer possessed both deletions (which explains the discrepancy between number of deletions [21] and individuals [20]). All but one white-tailed deer with either deletion were WT/WT for PRNP (the remaining deer was homozygous for S96 G). Overall, nine individuals with either deletion were negative for CWD and 11 were positive (the individual with both deletions was positive for CWD). We found no deletions in mule deer. There was no relationship between the occurrence of either deletion, promoter sequence variation (e.g., presence of consistent mutations), PRNP genotype and the presence of CWD. None of the individuals with 95 H possessed a deletion in the promoter, and no individual had both alleles 95 H and 96S. Deer classified as positive for CWD showed a range of ELISA-OD values, illustrating the quantitative nature of the diagnoses (Figure 2).

**Table 2.** PRNP genotype and presence of indels in white-tailed deer. See legend to Table 1.

allele 1	allele2	Indel 1 (TATA)	Indel 2 (TAAACAGA)	CWD Status	No. individuals
DQGASQ	DQGASQ	a/a		neg	2
DQGASQ	DQGASQ	A/a		neg	4
DQGASQ	DQGASQ	A/a		pos	7
DQGASQ	DQGASQ		b/b	neg	1
DQGASQ	DQGASQ		B/b	neg	2
DQGASQ	DQGASQ		B/b	pos	3
DQGGSQ	DQGASQ		B/b	pos	2
DQSASQ	DQSASQ	A/a		pos	1

We detected relatively low frequencies of the amino acid residues at positions 20, 95, 96, 103, and 225 that are thought to be related to decreased CWD susceptibility, with the exception of the Key deer, in which all 15 individuals were (homozygous) 96SS. Genotype 225 F was not found in the black-tailed deer samples from California or Alaska. Coues deer possessed a genotype (103 T) at a frequency of 0.26 (12 heterozygotes, 4 homozygotes); no other group possessed this genotype. Allele 96S was found in white-tailed deer from New York, and Minnesota; the 95 H allele was not found in Minnesota, New York, Key deer or Coues deer. We found greater levels of genetic variation (number of substitutions, haplotype diversity, nucleotide diversity) at the PRNP locus in white-tailed deer relative to mule deer and black-tailed deer (Table 3), although the sample of black-tailed deer from Alaska was invariant. Key deer showed similar levels of variation at PRNP to those found in white-tailed deer from New York.

**Table 3.** Summary of variation at the PRNP locus and part of its promoter in North American deer. 2 N = number alleles (2x number of individuals), S = number of segregating sites, Hd = haplotype diversity, k = average number of differences between alleles, Pi = nucleotide diversity, F(x) = frequency of amino acid residues thought to be associated with decreased susceptibility to CWD at positions 95, 96, 225, 103, and 20. Hybrid white-tailed X mule deer were excluded from this table.

PRNP	Wtde-NE	Mule-NE	Key	Btde-CA	Btde-AK	Wtde-NY	Coues	Wtde-MN
2 N	406	274	30	22	20	14	70	30
S	8	9	2	4	0	3	6	6
# alleles	10	12	2	5	1	4	7	8
Hd	0.446	0.583	0.4	0.47	0	0.74	0.62	0.8
k	0.554	0.774	0.81	0.53	0	0.95	1.43	1050
Pi	0.0007	0.0010	0.001	0.0007	0	0.0012	0.0019	0.00195
F(95H)	0.02	0	0	0	0	0	0	0
F(96S)	0.16	0	1.0	0	0	0.14	0	0.30
F(225 F)	0	0.047	0	0	0	0	0	0
F(103 T)	0	0	0	0	0	0	0.26	0
F(20 G)	0.014	0.078	0	0	0	0	0	0
Promoter								
2 N	338	242	30	20	12	12	70	30
S	23	5	9	5	0	10	7	17
# alleles	22	7	6	5	1	5	7	10
Hd	0.835	0.360	0.691	0.663	0	0.803	0.499	0.885
k	2.69	0.39	3.78	1.006	0	3.667	0.617	4.52
Pi	0.0053	0.0008	0.0073	0.00198	0	0.0072	0.0012	0.0089
8-base indel copies	9	0	0	0	0	0	0	0
4-base indel copies	15	0	0	4	0	0	67	1

We found numerous single-site polymorphisms in the promoter sequence with the exception of black-tailed deer from Alaska: white-tailed deer (Nebraska – 23, Minnesota, – 17, New York – 10; and all three states combined – 28), Coues deer (7), Key deer (9), black-tailed deer (CA – 5, AK – 0) and mule deer (NE – 5). One SNP occurred in the 8-base pair deletion, which was also reported by Heaton et al. [24]. We found 19 of the 21 sites identified by Heaton et al. [24] to be polymorphic (bp positions 290, 281 invariant), and an additional 18 sites that were polymorphic in our sample but not Heaton et al. [24] (base positions 128,150,166,184,237,241,245, 249,254,258,269,343,368,387,415,426,430, 479).

All deer surveyed here and by Vázquez-Miranda and Zink [14] have three tandem octapeptide repeats (PHGGGWGQ) starting at amino acid position 63, with no polymorphism observed.

## Discussion

Several aspects of variation in the PRNP gene and its promoter could influence progression of prion disease. Our study is the first to our knowledge to describe the relationship between PRNP genotype, indels in the promoter region, frequency of octarepeats, and the presence of CWD in white-tailed deer and mule deer. In bovines, deletions in the promoter are associated with an enhanced frequency of BSE, presumably due to an altered rate of transcription caused by loops in the promoter [18,30]. Heaton et al. [24] found that 6% of white-tailed deer and 2% of mule deer were heterozygous for an 8-base pair deletion, whereas from a considerably larger sample size, albeit from a different geographic region, our frequencies were 2.4% and 0%, respectively. We found no relationship between the presence of indels and the incidence of CWD in white-tailed deer (Table 2) from Nebraska, and indels were uncommon (~9% of sampled white-tailed deer and 0% of mule deer had an indel). The most common deletion removes a TATA box, which is important for the start of transcription, and this could explain its rarity. There was also no correlation between different PRNP genotypes and the two deletions observed in 19 white-tailed deer, which in general occurred in the most common genotypes. Thus, deletions in this promoter region do not appear to be associated with an enhanced incidence of CWD in our sample of white-tailed deer.

The inference of reduced susceptibility to CWD in deer possessing alleles 95 H and 96S comes from relatively few deer that were kept in captivity and inoculated with unnaturally concentrated doses of prions from later-stage CWD positive deer [8,9]. Individuals with alleles 95 H and 96S (either heterozygous or homozygous) lived longer, but still perished from CWD. It is unknown whether the onset of CWD in 95 H and 96S deer was shifted later in life or developed more slowly than other genotypes, and when during the course of infection these deer shed prions in sufficient quantity to infect healthy deer. Haley et al. [13] studied PRNP genotypes and presence of CWD in captive deer herds from the US and Canada, and found that 95 HH (n = 2) and 95 HQ/96SG (4) were negative for CWD, and that seven of 28 95 HQ/96 GG individuals were CWD positive. Haley et al. [13] found that 25 of 143 (17.5%) 96SS individuals were positive for CWD. Of interest is that Haley et al. [13] found that in Canada, only two of 42 116 GG and 96SG/116 GA genotypes were CWD positive, whereas 96 GG/116 GA exhibited 40 out of 189 deer that were positive for CWD. However, we found that only one of nine 95 H individuals was CWD positive, which was significant in an odds ratio test (P = 0.051; Table 4) relative to the wild type, a result also found by other studies [13,31].

**Table 4.** Likelihood and odds ratios of disease for a given genotype. Odds ratios are relative to the most common genotype (the wildtype), which is not necessarily the genotype with the highest risk for disease. An odds ratio of 1 indicates the reference genotype to which the other two are compared. Some combinations are not included because they were either not found or too rare for statistical analysis.

Allele	Total F	Neg F	Pos F	Odds	Confidence Interval	P-value
20D	0.90	0.44	0.47	2.41	1.007–5.743	0.048*
20 G	0.10	0.07	0.03	0.42	0.174–0.993	0.048*
95Q	0.98	0.49	0.49	7.96	0.986–64.239	0.051
95H	0.02	0.02	0.00	0.13	0.002–1.004	0.051
96G	0.83	0.42	0.40	0.95	0.569–1.596	0.855
96S	0.17	0.09	0.09	1.05	0.627–1.756	0.855
116A	0.97	0.50	0.47	0.54	0.156–1.875	0.332
116G	0.03	0.01	0.02	1.85	0.533–6.423	0.332
225S	0.96	0.48	0.48	0.84	0.250–2.822	0.778
225F	0.04	0.02	0.02	1.19	0.354–4.001	0.778
226Q	1.00	0.51	0.49	0.96	0.059–15.471	0.978
226K	0.00	0.00	0.00	1.04	0.065–16.753	0.978

In contrast to other studies, we found that individuals with or without the 96S allele were equally likely to be CWD positive. Although in previous studies, deer with a least one copy of the 96S allele experienced increased longevity (or delayed onset), the ages of our deer are unknown, and it is possible that many were relatively older bucks. ELISA tests are likely incapable of detecting CWD in very early disease stages. We found that having one copy of A116 G did reduce the odds of disease, but not significantly so, constituting only equivocal support for results in Haley et al. [13]. In a captive herd of white-tailed deer from Nebraska, O’Rourke et al. [5] found 13 CWD-positive deer (out of 133) that were heterozygous for the 116 G allele, also suggesting that the allele does not provide resistance to CWD. Thus, it is possible that a population with a high frequency of H95Q, S96G or A116 G alleles might show increased longevity, perhaps even mimicking the normal mortality schedule of past CWD-free generations. Haley et al. [13] suggested that captive breeding of individuals with 96S and 95H alleles might yield a relatively CWD-resistant herd. At the least, it could shift the occurrence of the disease past the first year or two of reproduction. It is noteworthy, however, that our percentages of CWD positive 96SS and 116 GA deer differed considerably from the results of Haley et al. [13], and suggests more widespread geographic testing is needed to verify the effects of particular genotypes on CWD susceptibility.

Our sequence data for deer outside of Nebraska came from individuals of unknown CWD status; however, CWD is not present in Florida (Key deer) or Arizona (Coues deer). Our sample of Key deer was fixed for 96S; these homozygous 96SS individuals might be relatively resistant should CWD make it to the Florida Keys. Our sample of Coues deer showed a relatively high frequency of 103 T, which has not been found in other white-tailed deer samples; this might be associated with reduced susceptibility to CWD, but this is unknown. However, we provide the first information at the PRNP locus, its promoter, and the number of octarepeats for these populations. Our widely spaced samples underscore the need for a denser sampling of deer throughout North America to document the pattern of genetic variation at the PRNP locus and its promoter as the incidence of CWD continues to increase geographically.

## Methods and materials

### Tissue collection and processing

Retropharyngeal lymph nodes collected from hunter-killed deer were tested by Enzyme-Linked Immunosorbent Assay (ELISA) using the standard protocol approved by the USDA at the University of Nebraska-Lincoln Veterinary Diagnostic Centre. ELISA assay was conducted using a commercial Transmissible Spongiform Encephalopathy Antigen Test kit (Bio-Rad) (Bovine Obex or Mule Deer/White-Tailed Deer/Elk Retropharyngeal Lymph node and Obex), following manufacturer’s instruction. Identification of the presence of CWD is based on optical density (OD) value that is equal to or greater than the USDA cut-off value (0.035). These initial positive samples were retested in duplicated samples or confirmed by immunohistochemistry (IHC). We compared the ELISA OD-values for each genotype. We did not attempt to determine the stage of infection, which requires inspection of sections of the obex.

### Study population

**Figure 1.**
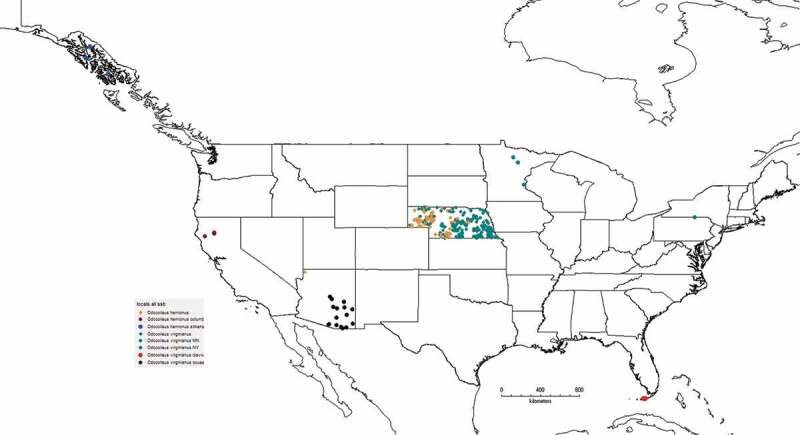
Distribution of deer samples used in this study.

To test the relationship between genotype and presence of CWD, we included 204 white-tailed deer (*Odocoileus virginianus*) and 141 mule deer (*O. hemonius*) that were collected in Nebraska (Figure 1); 90% of the deer were males. Of these, 66 mule deer were positive for CWD and three that were not definitively diagnosed, whereas 98 white-tailed deer tested positive and two were not definitively diagnosed. To provide information from populations that have not yet been sampled, we included white-tailed deer from Minnesota (n = 15), white-tailed deer from New York (n = 7), Key deer from Florida (n = 15), black-tailed deer from California (n = 11) and Alaska (n = 10), and Coues deer from Arizona (n = 35). These samples are not meant to provide a detailed examination of continental patterns, but are the first comparisons of the frequency of PRNP and promoter genotypes from these areas.

**Figure 2.**
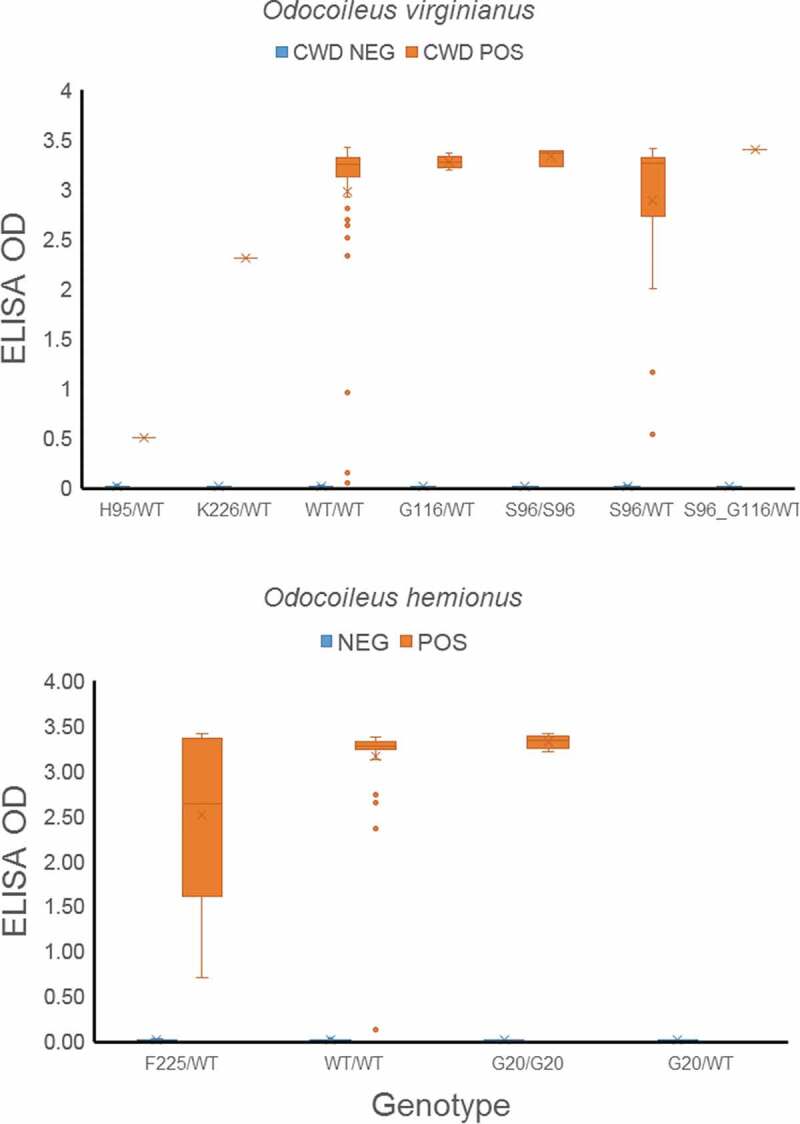
Plot of ELISA OD values by genotype for white-tailed deer and mule deer.

## PRNP and promoter sequencing

Our methods for sequencing a 771 bp segment of the PRNP gene followed Vázquez-Miranda and Zink [14]. We used primers TGTAAAACGATGGCCAGTCTCACCATTTCAGAATACCTC (forward or sense) and CAGGAAACAGCTATGACGGCCCTACATCATTCCTCA (reverse or antisense) to amplify 562 bp of the promoter gene, using the same PCR conditions as for the PRNP gene segment, and had the amplicons sequenced via Sanger sequencing at Rapid Genomics. Sequences were trimmed for quality, aligned, and based-called automatically in SEQUENCHER v5.4 [32]. Heterozygotes were identified using the ‘call heterozygous bases’ tool at 35% and confirmed by eye. Spectrograms were decomposed and indels identified with the ‘find indels’ tool in CODONCODE v9.0 (URL: https://www.codoncode.com/aligner/new.htm; accessed 30 June 2020). For both gene regions, we used DnaSP [33] to phase alleles. We computed the likelihood of odds ratios following Haley et al. [13]. We compared our PRNP alleles with those from a Blast search of white-tailed and mule deer.

We used logistic regression to identify alleles more or less likely to result in disease. Odds ratios, confidence intervals, likelihoods, and p-values were calculated in R (v 3.6.1). All statistics were calculated with the wildtype (DQGASQ/DQGASQ) as the reference allele or genotype. Genbank accession numbers are: PRNP (MT709334-MT710221) and promoter (MT722975-MT723742).
